# Concluding Notes on a Case of Splenomegalic Cirrhosis in a Child Aged Seven Years

**Published:** 1907-03

**Authors:** Edward Cecil Williams

**Affiliations:** Physician to the Royal Hospital for Sick Children and Women, Bristol.


### CONCLUDING NOTES ON A CASE OF SPLENOMEGALIC CIRRHOSIS IN A CHILD AGED SEVEN YEARS.

BY

EDWARD CECIL WILLIAMS, M.B. Cantab.,
*Physician to the Royal Hospital for Sick Children and Women, Bristol.*

TWELVE months ago I showed before this Society a boy, aged six years, with enlargement of liver and spleen, slight jaundice, stunted growth, and clubbing of fingers and toes. Abdomen was enlarged, surface veins distended. Liver dulness extended from the sixth rib to the level of the umbilicus. Spleen also much enlarged, extending within a couple of fingers' breadth of the iliac crest in the mid-axillary line; its notch could be felt on the outer border within 2½ to 3 inches of the middle line; it was dense and smooth. No evidence of ascites, no œdema of legs, but on each leg a few petechiæ. There was nothing distinctive in the blood picture. I regarded the clinical signs as falling in with the juvenile group of cases described by Gilbert and Fournier. On March 7th, 1906, the boy was discharged improved—no jaundice, liver not diminished in size, spleen certainly smaller. His motions had averaged one or two daily; they occasionally contained blood. The urine on only one occasion contained albumin. The boy was readmitted on May 9th, 1906. Liver was two inches below costal margin and was felt to be nodular. Spleen as before. Towards the end of June he was not so well, had slight rises of temperature, was drowsy, and abdomen began to fill. There was hæmaturia and œdema of legs, with blood in the motions. His mouth was ulcerated, and there were petechiæ on the legs. During the month of July paracentesis abdominis was performed three times, and 4½, 3½ and 1¾ pints of fluid were withdrawn, which afforded the patient much relief. The rises of temperature and drowsiness, with petechiæ and pain in the left side, seemed to denote an extra dose of poison in the system. On August 8th there was a slight reaccumulation of fluid, and 12 ounces were withdrawn. During September the child gradually lost ground. On October 2nd 36 ounces were withdrawn ; child was very anæmic, with a murmur over the mitral area ; skin again yellow, with traces of bile in the urine. About a month before his death he suffered from diarrhœa, with profuse watery motions, sometimes bloodstained. The motions averaged six, seven, and occasionally more a day. There was no further accumulation in his abdomen until a few days before his death, on November 27th. Leucin and tyrosin were not found in the urine. There was never any reason to suspect alcoholism, nor were there any signs of congenital syphilis. There was a doubtful history of paternal syphilis.

It is difficult to say what amount of splenic enlargement justifies the use of the word "splenomegaly." In my case the spleen seemed, when the child was first admitted, to be of excessive size. There is no doubt the spleen did diminish in size as the disease progressed. At the autopsy the spleen was found to weigh nine ounces, which is three times the average weight of a child aged seven years. The liver weighed 1 lb. 9 oz. Macroscopically it looked markedly cirrhotic, while microscopically a coarse, multilobular cirrhosis was the outstanding feature, with some increase in the number of bile-ducts. This condition cannot have anything to do with Banti's disease, which is the termination of the splenic anæmia of adults in multilobular cirrhosis and ascites. The second dentition is the earliest age at which the splenic anæmia of adults occurs, and the splenic anæmia of infants is a totally distinct affection, which seldom occurs after the age of two years. The poison may be manufactured in the intestine and conveyed to the liver by the portal vein, and thence to the spleen, or it may be introduced into the systemic circulation and have a selective affinity for the liver and spleen. There is no history of any infectious disease in my case as in some of the cases recorded by other observers.

The notes on the *post-mortem* appearances by Dr. J. M. Fortescue-Brickdale were as follows :—

The child was emaciated and jaundiced. The thyroid was normal, the thymus was fibrotic and wasted. There were extensive pleural and pleuro-pericardial adhesions, especially on the left side in front. The left pleural cavity was, however, not completely obliterated, and contained some milky fluid, which showed no fat globules under the microscope, and coagulated readily on heating. The heart was normal; the lungs somewhat œd matous. There was a considerable amount of ascites. The small intestines were normal; there was a small recent ulcer in the cæcum, and the colon appeared thickened and œdematous. The liver weighed 1 lb. 9 oz.; it was "hobnailed" in appearance, and the left lobe was especially shrunken and fibrotic. The spleen was firm and dark; it weighed 9 oz. There was perihepatitis and perisplenitis. The kidneys were normal.

Microscopically the spleen showed some congestion of the Malpighian follicles and thickening of the capsule and trabeculæ; the liver showed a cirrhosis of multilobular type, and in places there were collections of bile-ducts in the fibrous tissue bands, but it was difficult to say that any definite increase in these existed.

From the pathological appearances it was not possible to say which organ, liver or spleen, was first affected. There was nothing inconsistent with the diagnosis of splenomegalic cirrhosis of infantile type, as may be seen by reference to the published reports of cases. Dr. Taylor's case[1] gives a very close parallel, and in Dr. Parkes Weber's case[2] a similar coarse fibrosis of the liver existed. The spleen in the present instance, though enlarged, was certainly smaller than in most recorded cases; but in Gilbert's paper,[3] cases in which the spleen is only moderately enlarged are definitely included in the group.

There was no pathological evidence of syphilis, unless we are prepared to say that *any* case of obscure fibrosis of the liver is due to this cause. The number of bile-ducts and the character of the fibrosis were not such as to suggest Hanot's disease, and, moreover, there was no leucocytosis during life.

[1] *Guy's Hosp. Rep.*, 1897, liv. 1.

[2] *Tr. Path. Soc. Lond.*, 1895, xlvi. 71. [3] *Semaine méd.*, 1900, p. 186.

